# The outbreak of SARS-CoV-2 pneumonia calls for viral vaccines

**DOI:** 10.1038/s41541-020-0170-0

**Published:** 2020-03-06

**Authors:** Weilong Shang, Yi Yang, Yifan Rao, Xiancai Rao

**Affiliations:** Department of Microbiology, College of Basic Medical Sciences, Army Medical University (Third Military Medical University), 400038 Chongqing, China

**Keywords:** Vaccines, Microbiology, Pathogenesis, Infection, Vaccines

## Abstract

The outbreak of 2019-novel coronavirus disease (COVID-19) that is caused by SARS-CoV-2 has spread rapidly in China, and has developed to be a Public Health Emergency of International Concern. However, no specific antiviral treatments or vaccines are available yet. This work aims to share strategies and candidate antigens to develop safe and effective vaccines against SARS-CoV-2.

An outbreak of 2019-novel coronavirus (SARS-CoV-2) that causes atypical pneumonia (COVID-19) has raged in China since mid-December 2019 and has spread to 26 countries (February 20, 2020). The epidemic was identified by the first four cases confirmed on December 29, 2019 and was traced to the Huanan Seafood Wholesale Market, Wuhan city, Hubei Province, China^1^. A total of 75,465 cases with SARS-CoV-2 infections have been confirmed up to date (February 20, 2020), and 2,236 people have died in China^2^. COVID-19 spreads rapidly by human-to-human transmission with a median incubation period of 3.0 days (range, 0 to 24.0), and the time from symptom onset to developing pneumonia is 4.0 days (range, 2.0 to 7.0)^3^. Respiratory droplets and direct contact are conventional transmission routes for SARS-CoV-2, and fecal-to-oral transmission might also have a role^3^. Fever, dry cough, and fatigue are common symptoms at onset of COVID-19^4^. Most patients have lymphopenia and bilateral ground-glass opacity changes on chest CT scans^4,5^. No specific antiviral treatments or vaccines are available because it is a new emerging viral disease. Development of SARS-CoV-2-based vaccines is urgently required.

The entire virus particle-based preparation of vaccines, including inactivated and attenuated virus vaccines is advisable, because it is based on previous studies about the prevention and control of seasonal influenza vaccines^6^. The first SARS-CoV-2 (Wuhan-Hu-1) was successfully sequenced and its genomic sequence submitted to GenBank on January 5, 2020 (Accession no. MN908947.3)^7^. Subsequently large-scale culture of SARS-CoV-2 was quickly performed, and an inactivated virus vaccine could be prepared through the employment of established physical and chemical methods such as UV light, formaldehyde, and β-propiolactone^8^. The development of attenuated-virus vaccines is also possible by carefully screening the serially propagated SARS-CoV-2 with reduced pathogenesis such as induced minimal lung injury, diminished limited neutrophil influx, and increased anti-inflammatory cytokine expressions compared with the wild-type virus^9^. Both inactivated and attenuated virus vaccines have their own disadvantages and side effects (Table 1). Alternatively, new vaccine designs based on the putative protective antigen/peptides derived from SARS-CoV-2 should be considered.

**Table 1:** Advantages and disadvantages of different vaccine strategies.

Vaccine strategy	Advantages	Disadvantages	References
Inactivated virus vaccines	Easy to prepare; safety; high-titer neutralizing antibodies	Potential inappropriate for highly immunosuppressed individuals	^25^
Attenuated virus vaccines	Rapid development; induce high immune responses	Phenotypic or genotypic reversion possible; can still cause some disease	^25^
Subunit vaccines	High safety; consistent production; can induce cellular and humoral immune responses; high-titer neutralizing antibodies	High cost; lower immunogenicity; require repeated doses and adjuvants	^12,14^
Viral vector vaccines	Safety; induces high cellular and humoral immune responses	Possibly present pre-existing immunity	^12^
DNA vaccines	Easier to design; high safety; high-titer neutralizing antibodies	Lower immune responses in humans; repeated doses may cause toxicity	^23^
mRNA vaccines	Easier to design; high degree of adaptability; induce strong immune responses	Highly unstable under physiological conditions	^23^

Accumulated releases of SARS-CoV-2 genomes such as GenBank accession numbers MN908947.3, MN975262.1, NC_045512.2, MN997409.1, MN985325.1, MN988669.1, MN988668.1, MN994468.1, MN994467.1, MN988713.1, and MN938384.1 facilitate the development of virus-based subunit vaccines. SARS-CoV-2, which is similar to SARS-CoV and Middle East respiratory syndrome coronavirus (MERS-CoV), is an enveloped, single- and positive-stranded RNA virus with a genome comprising 29,891 nucleotides, which encode the 12 putative open reading frames responsible for the synthesis of viral structural and nonstructural proteins^7,10^. A mature SARS-CoV-2 has four structural proteins, namely, envelope (E), membrane (M), nucleocapsid (N), and spike (S)^10^. All these proteins may serve as antigens to stimulate neutralizing antibodies and increase CD4^+^/CD8^+^ T-cell responses^8,9^. However, subunit vaccines require multiple booster shots and suitable adjuvants to work, and certain subunit vaccines such as hepatitis B surface antigen, PreS1, and PreS2 may fail to yield protective response when tested clinically^11^. The DNA and mRNA vaccines that are easier to design and proceed into clinical trials very quickly remain experimental. The viral vector-based vaccines could also be quickly constructed and used without an adjuvant^12^. However, development of such vaccines might not start until antigens containing the neutralizing epitopes are identified^8^.

The E and M proteins have important functions in the viral assembly of a coronavirus, and the N protein is necessary for viral RNA synthesis^13^. Deletion of E protein abrogated the virulence of CoVs, and several studies have explored the potential of recombinant SARS-CoV or MERS-CoV with a mutated E protein as live attenuated vaccines^13,14^. The M protein can augment the immune response induced by N protein DNA vaccine against SARS-CoV;^15^ however, the conserved N protein across CoV families implies that it is not a suitable candidate for vaccine development, and the antibodies against the N protein of SARS-CoV-2 do not provide immunity to the infection^16^. The critical glycoprotein S of SARS-CoV-2 is responsible for virus binding and entry^16^. The S precursor protein of SARS-CoV-2 can be proteolytically cleaved into S1 (685 aa) and S2 (588 aa) subunits^10^. The S2 protein is well conserved among SARS-CoV-2 viruses and shares 99% identity with that of bat SARS-CoVs^10^. The vaccine design based on the S2 protein may boost the broad-spectrum antiviral effect and is worth testing in animal models. Antibodies against the conserved stem region of influenza hemagglutinin have been found to exhibit broadly cross-reactive immunity, but are less potent in neutralizing influenza A virus^17^. In contrast, the S1 subunit consists of the receptor-binding domain (RBD), which mediates virus entry into sensitive cells through the host angiotensin-converting enzyme 2 (ACE2) receptor^18^. The S1 protein of 2019-nCoV shares about 70% identity with that of human SARS-CoVs. The highest number of variations of amino acids in the RBD is located in the external subdomain, which is responsible for the direct interaction between virus and host receptor^10,18^. Blocking the initial entry of a virus is proposed as a successful strategy in controlling viral infection. Based on SARS vaccine development, most vaccine candidates target the S protein, which induces neutralizing antibody responses and stimulates a protective cellular immunity against SARS-CoVs^12^. Bukreyev et al.^19^ showed that immunization of African green monkeys with the full-length S protein of SARS-CoV protects monkeys from subsequent homologous SARS-CoV challenge. Administration of SARS-CoV RBD proteins can also induce highly potent neutralizing antibodies and long-term protective immunity in animal models^20^. Thus, the generation of antibodies targeting the S1 subunit of SARS-CoV-2 would be an important preventive and treatment strategy that can be tested further in suitable models before clinical trials^10^.

Vaccine delivery modality and immunization strategy are important issues to be considered for achieving effective antiviral immunity. As a cause of respiratory tract infection and as demonstrated by the findings of SARS-CoV-2 in stools^1,21^, administration of vaccines by oral or aerosol routes will induce mucosal immune responses and are possible modes of SARS-CoV-2 vaccine immunization. A safe DNA vector for preparation of DNA vaccines^22^, an attenuated virus strain for design of chimeric viral vaccines^23^, and engineered safe bacteria for production of membrane vesicle-vaccines^24^ could be explored for vaccine delivery and are worth investigating in the near future.

We can assume that virus-based vaccines should prove valuable in combatting COVID-19. In addition to the entire virus particle-associated inactivated or attenuated viral vaccines, the subunit candidates, such as S1 protein and/or the RBD element of SARS-CoV-2, are also valuable targets for vaccine design. Combining subunit vaccines with established or new adjuvants such as alum versus modern adjuvants such as the GSK AS series of adjuvants may represent a faster and safer strategy to move through early clinical development with the caveat that the protective efficacy may not be strong enough. As a result, immunizing the subunit vaccines with proper delivery platforms and immunization strategies to enhance the immune responses should be considered. We expect researchers who are racing against time will bring a new SARS-CoV-2-based vaccine from gene sequence to clinical testing in approximately 16–20 weeks.
